# TP53 regulates miRNA association with AGO2 to remodel the miRNA–mRNA interaction network

**DOI:** 10.1101/gr.191759.115

**Published:** 2016-03

**Authors:** Jonathan Krell, Justin Stebbing, Claudia Carissimi, Aleksandra F. Dabrowska, Alexander de Giorgio, Adam E. Frampton, Victoria Harding, Valerio Fulci, Giuseppe Macino, Teresa Colombo, Leandro Castellano

**Affiliations:** 1 Department of Surgery and Cancer, Imperial College London, Imperial Centre for Translational and Experimental Medicine, London W12 0NN, United Kingdom;; 2 Department of Cellular Biotechnologies and Hematology, Division of Molecular Genetics, “Sapienza” University of Rome, Rome 00161, Italy;; 3 Department of Surgery and Cancer, HPB Surgical Unit, Imperial College, Hammersmith Hospital Campus, London W12 0NN, United Kingdom;; 4 Institute for System Analysis and Computer Science “Antonio Ruberti,” National Research Council, Rome 00185, Italy;; 5 Department of Biotechnology, University of Verona, Verona 37134, Italy

## Abstract

DNA damage activates TP53-regulated surveillance mechanisms that are crucial in suppressing tumorigenesis. TP53 orchestrates these responses directly by transcriptionally modulating genes, including microRNAs (miRNAs), and by regulating miRNA biogenesis through interacting with the DROSHA complex. However, whether the association between miRNAs and AGO2 is regulated following DNA damage is not yet known. Here, we show that, following DNA damage, TP53 interacts with AGO2 to induce or reduce AGO2's association of a subset of miRNAs, including multiple let-7 family members. Furthermore, we show that specific mutations in TP53 decrease rather than increase the association of let-7 family miRNAs, reducing their activity without preventing TP53 from interacting with AGO2. This is consistent with the oncogenic properties of these mutants. Using AGO2 RIP-seq and PAR-CLIP-seq, we show that the DNA damage–induced increase in binding of let-7 family members to the RISC complex is functional. We unambiguously determine the global miRNA–mRNA interaction networks involved in the DNA damage response, validating them through the identification of miRNA-target chimeras formed by endogenous ligation reactions. We find that the target complementary region of the let-7 seed tends to have highly fixed positions and more variable ones. Additionally, we observe that miRNAs, whose cellular abundance or differential association with AGO2 is regulated by TP53, are involved in an intricate network of regulatory feedback and feedforward circuits. TP53-mediated regulation of AGO2–miRNA interaction represents a new mechanism of miRNA regulation in carcinogenesis.

DNA damage activates TP53, which acting principally as a transcription factor, directs DNA repair or, where irreparable damage has occurred, the initiation of programmed cell death. It does so by orchestrating the transcription of a number of messenger RNAs (mRNAs) (Menendez et al. 2009), noncoding RNAs—both long noncoding RNAs (lncRNAs) (Hung et al. 2011) and microRNAs (miRNAs)—which are involved in controlling these pathways (Krell et al. 2013). TP53 also modulates the nuclear biogenesis step of a group of miRNAs by interacting with the DROSHA complex through the DEAD box RNA helicase DDX5 (Suzuki et al. 2009). A number of chemotherapeutic agents with anti-cancer activity act as DNA damaging agents, including doxorubicin (DOX), which induces double-strand DNA breaks (DSBs) that activate TP53-mediated cell signaling pathways such as apoptosis, senescence, and cell cycle arrest (Krell et al. 2013; Kruiswijk et al. 2015). DOX is widely used to activate DNA damage in cell lines in order to study TP53 function.

Mutations in, or inactivation of, TP53 are the most frequent abnormalities observed in cancer cells (Hollstein et al. 1991). Furthermore, miRNAs are often dysregulated in cancer, indicating that refining our knowledge of their roles in TP53-signaling networks and their gene targets is crucial to achieving a greater understanding of tumorigenesis.

Importantly, miRNAs only become active regulators of their mRNA targets once they interact with AGO1-4 proteins, the key components of the RNA-induced silencing complexes (RISCs). When loaded with a miRNA, AGO proteins inhibit the expression of their targets, recognizing them through miRNA-target base-pairing (Bartel 2009).

Isolation and sequencing of RNAs (RNA-seq) that interact with AGO proteins has been widely performed to globally identify functional miRNA targets in vivo at single nucleotide resolution. These include photoactivatable-ribonucleoside-enhanced crosslinking and immunoprecipitation followed by deep sequencing (PAR-CLIP-seq), high-throughput sequencing of RNA isolated by crosslinking immunoprecipitation (HITS-CLIP), individual-nucleotide resolution crosslinking and immunoprecipitation (iCLIP), and finally, RNA immunoprecipitation followed by deep sequencing (RIP-seq) (Chi et al. 2009; Hafner et al. 2010; Konig et al. 2011; Schraivogel et al. 2011). More recently these techniques have been coupled with crosslinking, ligation, and sequencing of hybrids (CLASH), a technique involving a ligation reaction during the RNA preparation step to covalently join miRNAs to the RNA regions with which they interact (Helwak et al. 2013; Helwak and Tollervey 2014). Following this procedure, the bioinformatic isolation and analysis of the chimeric products allows the unambiguous identification of miRNA targets (Helwak et al. 2013; Grosswendt et al. 2014; Helwak and Tollervey 2014).

Importantly, despite that regulation of miRNA biogenesis has been extensively explored, both at the transcription and maturation levels, to the best of our knowledge, modulation of the association between miRNAs and the RISC complex upon cellular stimulation has not been previously demonstrated. In light of this, we hypothesized that modulation of miRNA association with AGO2 could be a further step in the regulation of miRNA activity upon specific cellular stimuli. Thus, we aimed to investigate whether such a mechanism might represent a novel TP53-mediated process, capable of regulating miRNA activity in a different manner to those previously described, and with relevance to the DNA-damage response and carcinogenesis.

## Results

### TP53 regulates the association between a subgroup of miRNAs and AGO2

To evaluate if DNA damage induced by DSBs affects the association between certain miRNAs and the RISC complex, we stimulated either *TP53*^+/+^ or *TP53*^−/−^ HCT116 human colon cancer cell lines with DOX for 24 h and performed small RNA-seq from total RNA and from RNA purified from immunoprecipitated AGO2 (AGO2-RIP-seq), analyzed using a tailored bioinformatics pipeline (Supplemental Fig. S1; Supplemental Methods). As illustrated in Supplemental Figure S2A, a number of miRNAs exhibited a statistically significant, TP53-dependent increase or decrease in cellular abundance (Benjamini-Hochberg adjusted Student's *t*-test *P*-value < 0.05) following DOX treatment. Here, we confirmed the TP53-dependent up-regulation of miR-34a, miR-143, and miR-107, consistent with previous findings (He et al. 2007; Suzuki et al. 2009) supporting the reliability of our approach (Supplemental Fig. S2A; Supplemental Table S1). However, a number of miRNAs demonstrated an increase or decrease in cellular abundance in both *TP53*^+/+^ and *TP53*^−/−^ cells following DOX treatment (Supplemental Fig. S2B; Supplemental Table S1), indicating that these expression changes occurred in a TP53-independent manner. Interestingly, our small RNA-seq approach identified, for the first time, that miR-3065-3p and miR-3065-5p are the miRNAs most strongly up-regulated by DNA damage (Supplemental Fig. S2B; Supplemental Table S1). This occurred in a TP53-independent manner (Supplemental Fig. S2B; Supplemental Table S1), indicating that these miRNAs could be central to the DNA damage response, and we validated this finding by RT-qPCR (Supplemental Fig. S2C).

**Figure 1.**
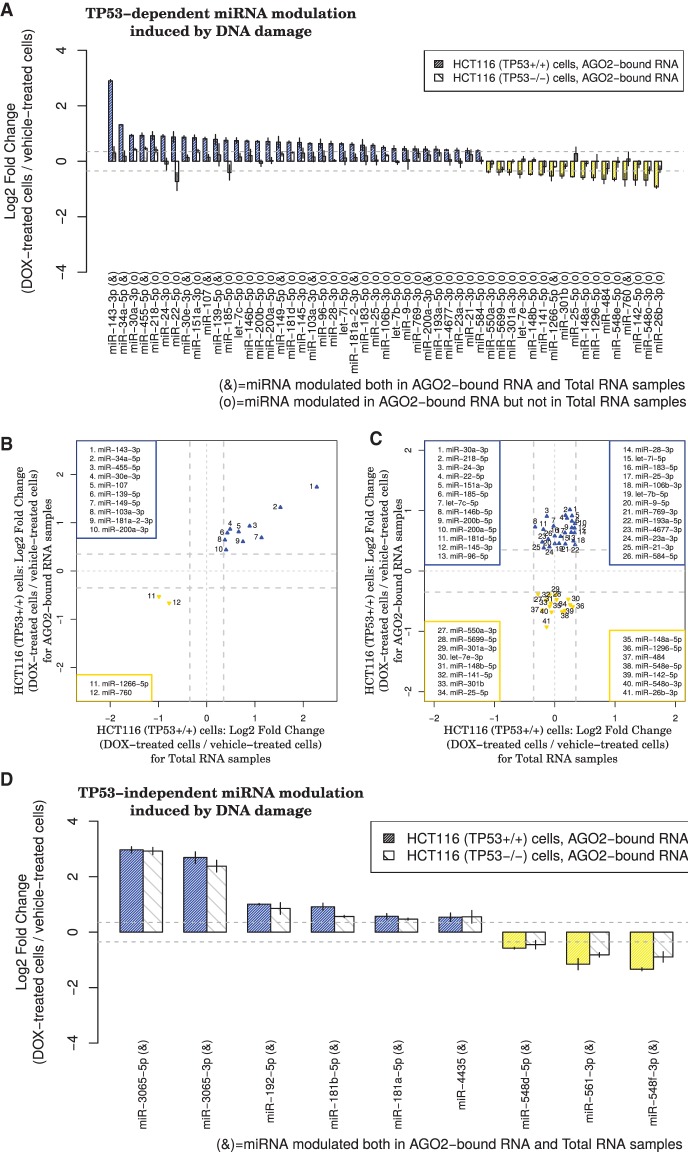
TP53 is required for DNA damage–induced modulation of the association between AGO2 and a subset of miRNAs. (*A*) Bar plot of fold changes for 53 miRNAs exhibiting statistically significant (Benjamini-Hochberg adjusted Student's *t*-test *P*-value < 0.05) differential expression of small RNAs isolated from immunoprecipitated AGO2 after DOX treatment. In some cases (12/53 miRNAs marked by an “&” symbol in the bar plot *x*-axis labels), the observed changes in levels of AGO2 binding reflect changes in abundance in total RNA induced by the treatment. In other cases (41/53 miRNAs marked by an “o” symbol in the bar plot *x*-axis labels), however, the observed changes in AGO2 binding occur in the absence of changes in abundance in total RNA. (*B*) Scatterplot showing miRNAs that change significantly in both the AGO2-immunoprecipitation and total RNA samples (HCT116 *TP53*^+/+^ cells). (*C*) Scatterplot showing miRNAs that significantly change in the AGO2-immunoprecipitation but not in the total RNA samples (HCT116 *TP53*^+/+^ cells). (*D*) Bar plot of fold changes for nine miRNAs exhibiting statistically significant (Benjamini-Hochberg adjusted Student's *t*-test *P*-value < 0.05) and TP53-independent changes in expression. For these miRNAs, changes in AGO2 binding reflect changes in abundance in total RNA (9/9 miRNAs marked by an “&” symbol in the bar plot *x*-axis labels). In *A* and *D*, each bar represents the mean and standard deviation of three biological replicates. In *B* and *C*, thicker dotted gray lines indicate the selected log-fold change cutoff (0.35), and thinner dotted gray lines indicate a log-fold change equal to 0.

Strikingly, although for many miRNAs, their increase in abundance on AGO2 correlated with an increase in cellular expression (marked “&”) (Fig. 1A,B), for a subset, DNA-damage modulated their association with AGO2 (Benjamini-Hochberg adjusted Student's *t*-test *P*-value < 0.05) but not their cellular abundance (marked “o” ) (Fig. 1A,C; Supplemental Fig. S3A). This occurred in a TP53-dependent manner (Fig. 1A,C) as no change in miRNA association with AGO2 was seen in HCT116 *TP53*^−/−^ cells (Fig. 1D). Interestingly, at least three members of the let-7 family exhibited such increases in AGO2 binding (Fig. 1A,C; Supplemental Fig. S3A) that were not due to changes in AGO2 expression (Supplemental Fig. S3B). To increase our confidence in defining the miRNAs whose abundance was regulated by DNA damage, we only considered those whose expression was changed in a comparable manner in both the AGO2-RIP-seq and the RNA-seq experiments (Fig. 1A, marked “&”; and Fig. 1B). Briefly, we identified 10 miRNAs that were up-regulated and 2 that were down-regulated in a TP53-dependent manner (marked “&”) (Fig. 1A,B), and six miRNAs that were up-regulated and three that were down-regulated independently of TP53 (marked “&”) (Fig. 1D). Furthermore, we identified 26 miRNAs whose association with AGO2 increased and 15 whose association with AGO2 decreased following DOX treatment, without their expression levels changing, finding that this only occurred in the presence of wild-type TP53 (marked “o”) (Fig. 1A,C; Supplemental Table S1).

### The association of AGO2 with let-7 family miRNAs is significantly increased in response to DOX in the presence of wild-type TP53 but is decreased in the presence of mutant TP53

**Figure 2.**
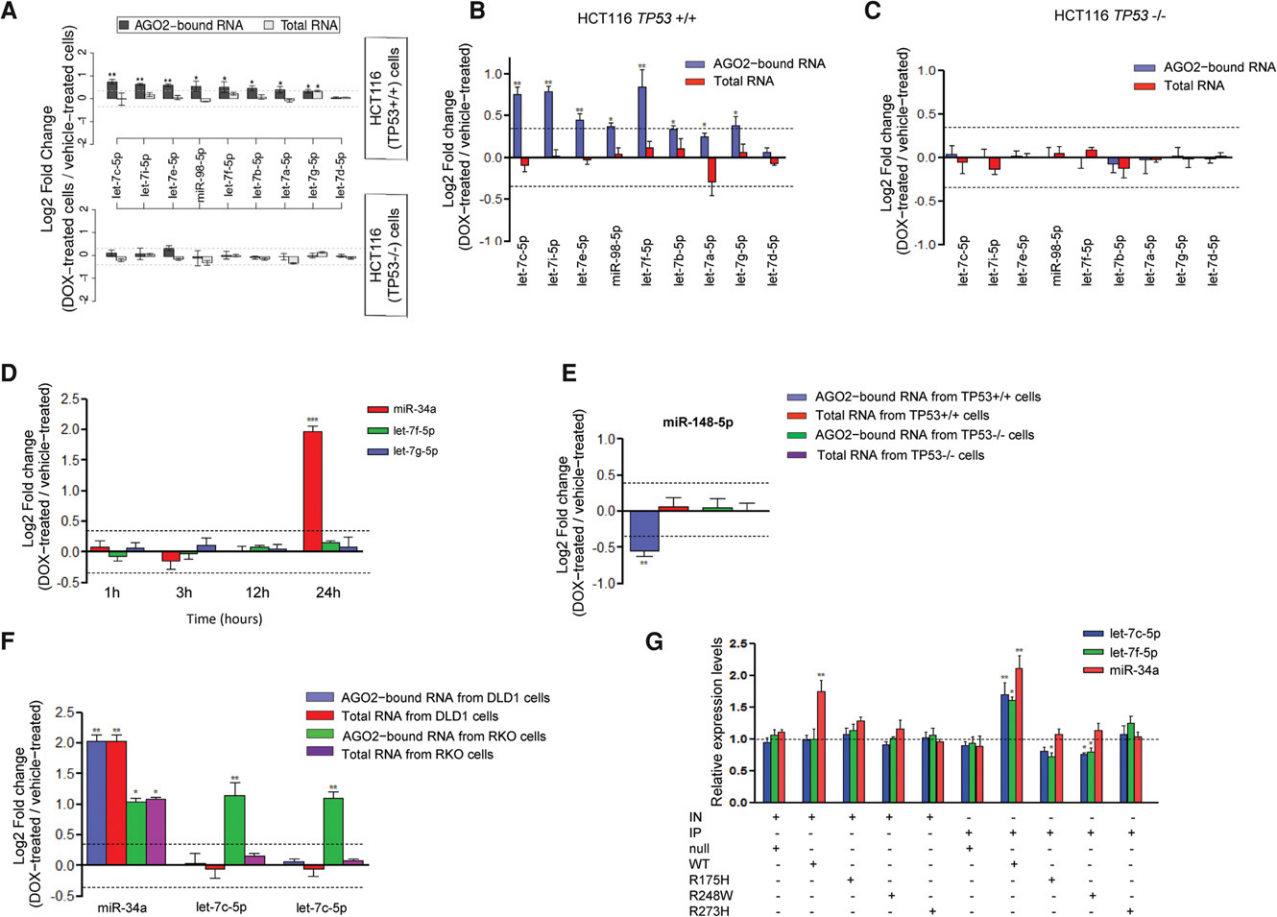
DOX-induced DNA damage induces TP53-dependent differential binding of specific miRNAs to AGO2. (*A*) Fold changes in let-7 family levels determined by a combined small RNA-seq and AGO2-RIP-seq approach (miRNAs demonstrating a positive or negative fold change equal to or greater than the selected cutoff of 0.35 [log_2_] and a *t*-test *P*-value < 0.05 after correcting for multiple testing with the Benjamini-Hochberg method [Benjamini and Hochberg 1995], were considered to be significant). (*B*,*C*) RT-qPCR analysis demonstrates that the binding of let-7 family members onto AGO2 is increased significantly in HCT116 *TP53*^+/+^ but not in *TP53*^−/−^ cells following DOX treatment. (*D*) Time course showing miR-34a and let-7 family fold changes following DOX-induced DNA damage in total RNA extracted from HCT116 *TP53*^+/+^ cells. (*E*) Fold change in miR-148-5p expression in total RNA or AGO2-bound RNA samples following an AGO2 IP in DOX- or vehicle-treated HCT116 *TP53*^+/+^ and *TP53*^−/−^ cell lines. (*F*) Fold change in let-7 and miR-34a expression following an AGO2 IP in DLD1 and RKO cell lines following DOX-induced DNA damage. (*G*) Fold change in let-7 family and miR-34a expression following an AGO2 IP in HCT116 *TP53*^−/−^ cells transfected with plasmids expressing wild-type or mutant TP53 (R175H, R248W, R273H). (IN) RNA isolated from input samples; (IP) RNA isolated from the immunoprecipitated AGO2; (null) HCT116 *TP53*^−/−^ cells; (WT) HCT116 *TP53*^−/−^ cells transfected with the plasmid expressing wild-type TP53; (R175H) HCT116 *TP53*^−/−^ cells transfected with the plasmid expressing R175H TP53 mutant; (R248W) HCT116 *TP53*^−/−^ cells transfected with the plasmid expressing R248W TP53 mutant; (R273H) HCT116 *TP53*^−/−^ cells transfected with the plasmid expressing R273H TP53 mutant. At least three independent experiments have been performed in all cases.

The role of let-7 miRNAs in repressing cell cycle and cancer progression has been demonstrated by several independent groups (Boyerinas et al. 2010). However, their abundance has not been found to change in response to DSBs stimulated by DOX treatment in HCT116 cells in this (Figs. 1B,C; Supplemental Fig. S2A; Supplemental Table S1) or previous high-throughput studies (Chang et al. 2007; He et al. 2007; Suzuki et al. 2009). Strikingly, our results indicate a novel mechanism whereby, instead of modulating the cellular abundance of the let-7 family, DSBs increase the association between these miRNAs and AGO2. Having identified a TP53-dependent modulation of AGO2 binding for three members of the let-7 family and considering their roles in negatively regulating cell cycle progression, we subsequently examined expression levels of all let-7 family members in our small RNA-seq and RIP-seq data. Specifically, we compared the changes in the expression levels induced by DNA damage (i.e., log-fold changes of DOX-treated versus untreated samples) between total RNA and AGO-immunoprecipitated RNA data for both *TP53*^+/+^ and *TP53*^−/−^ cells. This revealed a significant TP53-dependent regulatory effect on the association between AGO2 and the entire let-7 family apart from let-7d (Fig. 2A), and we validated these findings by RT-qPCR (Fig. 2B,C). The cellular abundance of both let-7f and let-7g was unchanged during a time course experiment following 1–24 h of DOX treatment (Fig. 2D), indicating that the TP53-dependent increase in their association with AGO2 demonstrated after 24 h treatment was not due to an earlier and transient increase in let-7 expression within the treatment period. We also confirmed a decreased association between miR-148a-5p and AGO2 in response to DOX treatment (Fig. 2E), whereas no significant change in miR-148a-5p expression was observed in a time course experiment (Supplemental Fig. S3C). Strikingly, the association between let-7c or let-7i and AGO2 significantly increased in both HCT116 *TP53*^+/+^ (Fig. 2A,B) and wild-type RKO human colon cancer cell lines (Fig. 2F) but did not change in DLD1 human colon cancer cell lines that expressed a mutated form of TP53 (Fig. 2F). Reintroduction of wild-type TP53 into HCT116 *TP53*^−/−^ cells restored both the induction of miR-34a and the increased association between let-7 and AGO2 in response to DOX (Fig. 2G). Noticeably, the introduction of two mutant TP53 proteins (R175H and R248W) into HCT116 *TP53*^−/−^ cells, which have been shown to exert oncogenic instead of tumor suppressive activity (Liu et al. 2010), significantly reduced the association between let-7 and AGO2 (Fig. 2G), while a third TP53 mutant (R273H) lost its capacity to affect this association.

### TP53 interacts with AGO2 in both the cytoplasmic and nuclear compartments following DOX-mediated DSBs

**Figure 3.**
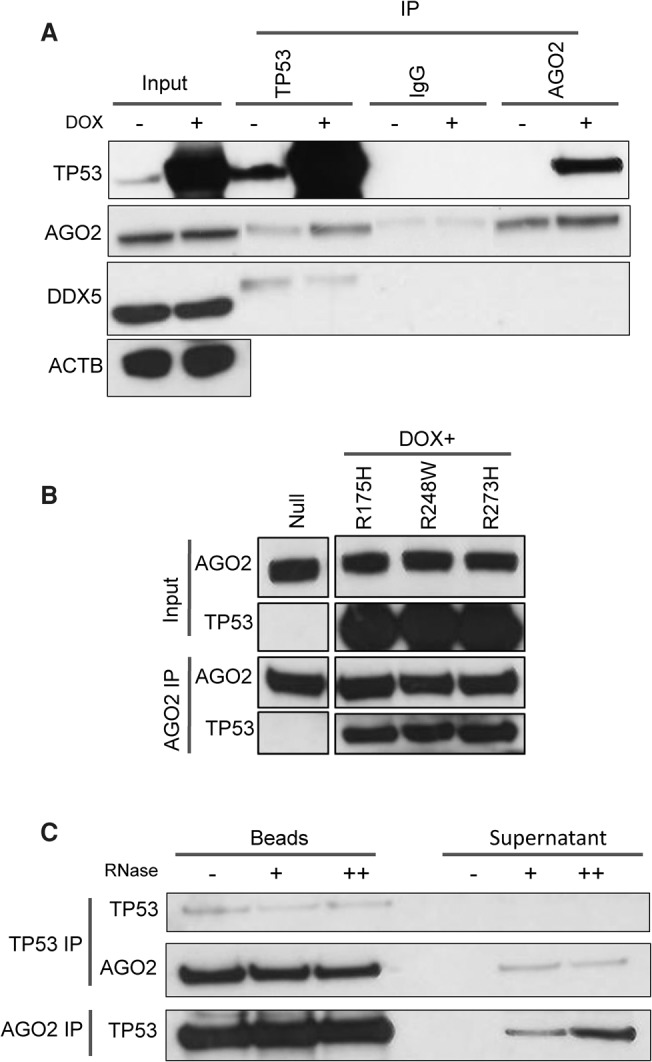
AGO2 interacts with TP53 in an RNA-dependent manner. HCT116 *TP53*^+/+^ were treated with vehicle or DOX for 12 h, and coimmunoprecipitation assays were then performed to determine whether an interaction exists between TP53 and AGO2 proteins. (*A*) Reciprocal co-IP experiments using AGO2 and TP53 antibodies demonstrate an interaction between the two proteins. DDX5 was used here as a positive control for the immunoprecipitation, and ACTB was used as a loading control housekeeping gene in the input sample. (*B*) To test the impact of mutations in TP53 on its ability to interact with AGO2, *TP53*^−/−^ cells were transfected with empty plasmid (null) or plasmids expressing mutated forms of TP53 (R175H, R248W, and R273H), treated with DOX for 12 h, and subjected to co-IP. (*C*) To investigate whether the association between TP53 and AGO2 is RNA-dependent, immunoprecipitates of AGO2 and TP53 from cells treated with DOX for 12 h were digested with increasing quantity of RNase A for 30 min at 37°C, separated into supernatant and beads, and analyzed by immunoblotting. At least three independent experiments have been performed in all cases.

**Figure 4.**
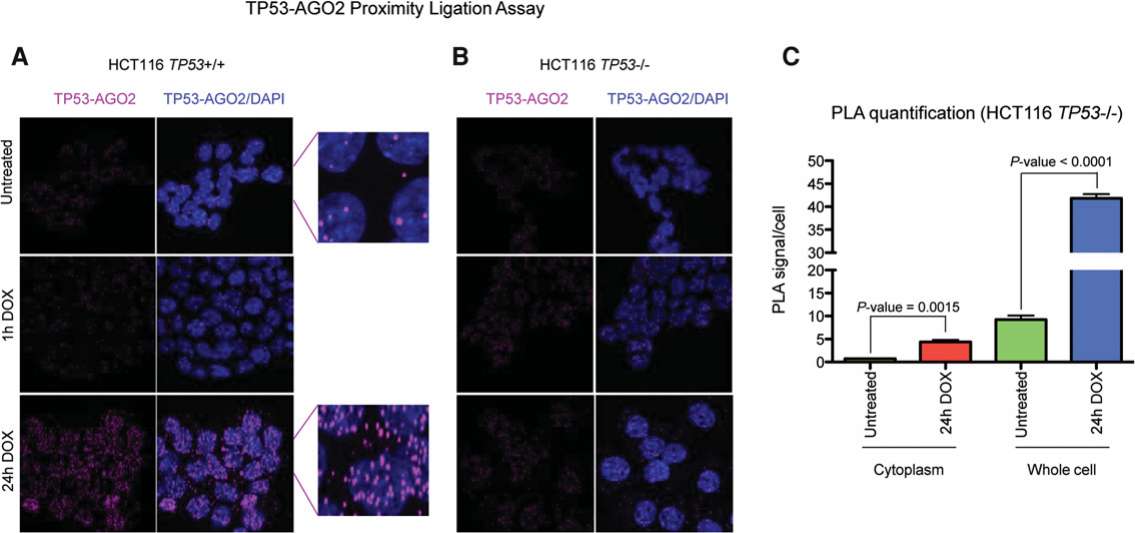
DNA damage increases the interaction between TP53 and AGO2 both in the cytoplasm and in the nucleus. (*A*) TP53-AGO2 PLA in HCT116 *TP53*^+/+^ cells shows that TP53 interacts with AGO2 after 24 h of DOX treatment, and this interaction also occurs in the cytoplasm but is predominantly observed in the nucleus. (*B*) Absence of green spots over background level in *TP53*^−/−^ HCT116 cells indicates that the interaction shown by the PLA in wild-type cells, after 24 h of DOX treatment (*A*) is specific. (*C*) Bar plot showing quantification of PLA spots in the cytoplasm and in the whole HCT116 wild-type cells. Slides were imaged using a Leica TCS SP5 confocal laser-scanning microscope and analyzed using ImageJ software. The number of PLA foci was manually counted in a blind fashion (100 cells per each condition in each of three independent experiments). Cytoplasmic foci were defined as fluorescent spots that did not colocalize with the nuclei stained with DAPI (two-tailed Student's *t*-test). At least three independent experiments have been performed in all cases.

Co-IP experiments indicated that wild-type and mutant TP53 variants all interact with AGO2 after DOX treatment (Fig. 3A,B). Interestingly, the interaction between AGO2 and TP53 was decreased after RNase treatment, alongside increases in the unbound supernatant (Fig. 3C), indicating that RNAs enhance the AGO2–TP53 association. The interaction between TP53 and AGO2 was previously demonstrated in a genome-wide association study in *Drosophila melanogaster* (Lunardi et al. 2010), demonstrating that this cobinding is highly conserved across species. Next, we used a proximity ligation assay (PLA) in untreated and DOX-treated cells to evaluate the cellular compartment in which this binding occurs, following DNA damage (Fig. 4A). We found that TP53 interacted with AGO2 after 24 h of DOX treatment (Fig. 4A), consistent with our demonstration of a TP53-dependent mechanism regulating the association between AGO2 and a subgroup of miRNAs (Fig. 1A,C). Interestingly, AGO2-TP53 complexes were present in both the cytoplasm and the nucleus following 24 h of DOX treatment (Fig. 4A,C), but the majority were located in the nucleus (Fig. 4A,C), indicating that additional functions may be exerted by this complex in the nuclear compartment. Accordingly, it has been previously demonstrated that DICER1- and DROSHA-dependent functional small RNAs arise from DSBs (Francia et al. 2012). This, coupled with our results, suggests that AGO2 may interact with this small RNA population in the nucleus. AGO2 was actively imported into the nucleus upon DOX treatment, independently of TP53 (Supplemental Fig. S4A,B). The absence of fluorescent spots over the background level in HCT116 *TP53*^−/−^ cells indicated the existence of a specific interaction (Fig. 4B). AGO2–TP53 binding occurred in response to DOX in both wild-type (Figs. 3A, 4A,C) and TP53 mutant expressing cells (Fig. 3B), but the TP53-dependent effect on the let-7's association with AGO2 was different in cells with mutant TP53 (Fig. 2G).

### A set of transcripts, whose cellular abundance does not change following the induction of DSBs, are differentially associated with AGO2

Next, we wished to determine the mRNA targets of the DNA damage–regulated miRNAs. It is known that TP53 directly activates the transcription of several genes (Wei et al. 2006) in response to DNA damage. However, very little is known about the fraction of genes that do not change at the transcriptional level but that are post-transcriptionally regulated in response to DNA damage. To identify genes that may be post-transcriptionally regulated through an AGO2–miRNA interaction, but are not transcriptionally modulated, we performed RNA-seq and AGO2-RIP-seq of polyadenylated transcripts (Supplemental Fig. S5; Supplemental Table S2). As expected, our RNA-seq showed extensive deregulation of genes involved in apoptosis, DNA damage response, and cell cycle (Supplemental Fig. S6A–F; Supplemental Table S3) as well as previously described TP53 targets (Supplemental Fig. S6A). Consistent with our previous findings (Krell et al. 2014), expression of snoRNAs derived from the *GAS5* locus was also increased via TP53 (Supplemental Table S2). Notably, by combining our RNA-seq and AGO2-RIP-seq data, we identified 144 and 161 genes, whose binding onto AGO2 increased and decreased, respectively, in response to DNA damage in both replicates, but whose expression showed no change in total RNA samples (Supplemental Fig. S6G), indicating that these genes are post-transcriptionally regulated through a DNA damage–mediated miRNA–AGO2-target interaction. A pathway enrichment analysis involving these two groups of genes did not show any significant category of gene enrichment when the *P*-values were adjusted for multiple hypothesis testing. Nevertheless, we noticed that among the transcripts that were more associated with AGO2 (indicative of increased repression by miRNAs) following DNA damage, at least eight code for proteins that negatively regulate apoptosis (Supplemental Fig. S7A). Conversely, among the transcripts less associated with AGO2 (possibly because their repression by miRNAs was released) in response to DSBs, there were at least nine that code for proteins involved in DNA repair (Supplemental Fig. S7B), demonstrating that this regulation might be important for the DNA damage response.

In addition, among the transcripts that were either more or less associated with AGO2 following DOX treatment, 31 are annotated as lncRNAs and 12 as pseudogenes (Supplemental Table S2), suggesting that competing activity between miRNAs and noncoding RNAs (Poliseno et al. 2010; de Giorgio et al. 2013) might occur during the DNA damage response. *NEAT1* was among the lncRNAs whose association with AGO2 was reduced by DNA damage (Supplemental Table S2). This lncRNA is essential for the formation of paraspeckles (Clemson et al. 2009), which have been implicated in cancer cell survival (Choudhry et al. 2015).

Importantly, transcripts that were more associated with AGO2 in response to DNA damage were significantly enriched for let-7 seed matches (Supplemental Table S4), indicating that the aforementioned TP53-induced association between let-7 and AGO2 is functional.

### PAR-CLIP-seq identifies the mRNA targets of those miRNAs modulated by DSBs

To identify the mRNA targets of those miRNAs modulated by DSBs, we treated HCT116 *TP53*^+/+^ and *TP53*^−/−^ cells with DOX and performed an AGO2 PAR-CLIP-seq experiment. After UV crosslinking, partial RNase digestion, and AGO2 IP, we prepared small RNA libraries from nine replicates per treatment. We used the PARalyzer tool (Corcoran et al. 2011) to identify genome-wide AGO2 interaction sites from our PAR-CLIP data based on the rate of T to C conversions and read density and obtained a total of 111,841 clusters of reads, representing AGO2 binding sites from all of the sequenced libraries (corresponding to a unique set of 54,256 sites). We then annotated the collection of AGO2 binding sites obtained to genomic regions to evaluate AGO2 binding distribution and observed a neat binding preference for 3′ untranslated regions (3′ UTRs) and coding DNA sequences (CDSs) (Supplemental Fig. S8A). In fact, it is known that miRNAs enact post-transcriptional repression of targets through interacting with coding CDSs (Tay et al. 2008) as well as 3′ UTRs and 5′ UTRs (Lytle et al. 2007). Since clusters of reads derived from PAR-CLIP are selected by identifying T to C conversion sites (see Methods), they disproportionately arise from T-rich regions. This indicates that the AGO2 distribution in the various parts of the genes needs to be adjusted for T frequency in PAR-CLIP analysis. To this end, we calculated the nucleotide frequency from 5′ UTR, CDS, and 3′ UTR sequence annotations used here to map the PAR-CLIP sequencing reads (Supplemental Fig. S8B). As expected, Ts were more enriched in the 3′ UTRs than the CDSs (T frequency equal to 29.68% and 21.78%, respectively) (Supplemental Fig. S8B), and we used their frequencies to normalize PAR-CLIP cluster distribution (Supplemental Fig. S8C). After normalizing for T content, we found that AGO2 was almost equally distributed between 3′ UTR and CDS regions (Supplemental Fig. S8C).

Subsequently, we analyzed occurrences of short sites matching the reverse-complement of miRNA canonical seed regions (Lewis et al. 2005), along with their evolutionary conservation, to assign AGO2 binding sites identified by the PAR-CLIP analysis to miRNAs. In accordance with previous findings (Hafner et al. 2010), the positional distribution of the first miRNA recognition element (MRE) nucleotide in each AGO2 binding site showed a preference for positions from −2 to +5 with respect to the predominant T to C transition within the clusters (Supplemental Fig. S9), and we used this information together with MRE conservation and length to prioritize the assignment of a single expressed miRNA to each AGO2 binding site where there was ambiguity due to multiple possible MREs (see Methods for further details). Since miRNAs also target genes in a “noncanonical” manner, through bulges and G:U pairs in the seed region (Chi et al. 2012; Helwak et al. 2013; Clark et al. 2014), and given our novel findings of an increased association between let-7 and AGO2 following DNA damage, we wished to complement our analysis by taking into account potential noncanonical interactions between let-7 and their gene targets on AGO2. To achieve this, we used the miRNA–mRNA interaction data from Clark and coworkers (available at https://cm.jefferson.edu/clip_2014/) that contains HITS-CLIP results from multiple cell lines (Clark et al. 2014), focusing on let-7 interactions and selecting those that overlapped with our AGO2 binding sites (Supplemental Table S5). Finally, based on the analysis of AGO2 binding sites and their assignment to targeting miRNAs, we built a comprehensive miRNA–mRNA interaction network (Supplemental Fig. S10). Noticeably, this miRNA-targetome network showed coregulation of genes important for cell cycle progression, including *CCND1* and *POGZ* that were targeted by multiple up-regulated miRNAs and by let-7 members (Supplemental Fig. S10; Supplemental Table S5). We validated by luciferase reporter assays the regulatory interaction of four different miRNAs (namely let-7a, let-7d, miR-23a, and miR-34) that appeared to putatively coregulate both *CCND1* and *POGZ* (Supplemental Fig. S11). Among these, the only experimentally validated interaction absent in the PAR-CLIP interaction network was the one between miR-34a and *POGZ*. Nevertheless, we also included this potential interaction in the reporter analysis because of the relevance of miR-34a for the DNA damage response (He et al. 2007) and because an 8-mer matching to the miR-34a seed in the *POGZ* 3′ UTR is conserved among vertebrates (Supplemental Fig. S11).

**Figure 5.**
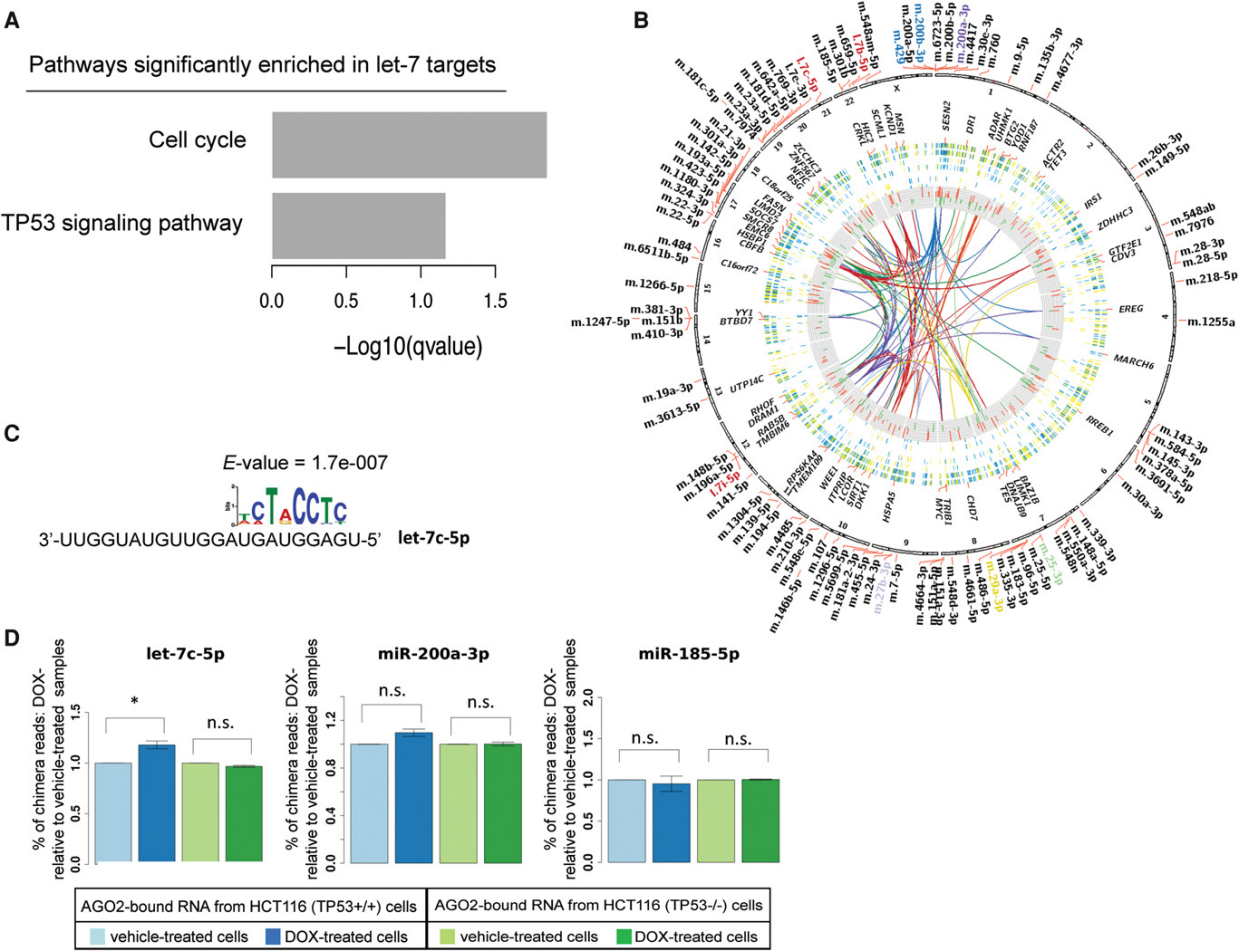
let-7-mRNA chimeras are significantly more abundant after DOX treatment in TP53 wild-type cells compared with untreated cells. (*A*) KEGG pathways enrichment analysis performed using clusterProfiler (https://www.bioconductor.org/packages/3.3/bioc/html/clusterProfiler.html) (Yu et al. 2012) for the list of genes included in the let-7 subnetwork. (*B*) Circos (http://circos.ca/) (Krzywinski et al. 2009) circularly layered heatmaps and histograms from different experiment data tracks produced in the present study mapped on the human genome (hg19). From *outer* to *inner* circle: heatmap of gene expression changes found in DOX-treated versus vehicle-treated *TP53*^+/+^ HCT116 cells by RNA-seq of total and AGO2-bound RNA samples; gene expression changes found in DOX-treated versus vehicle-treated *TP53*^−/−^ HCT116 cells by RNA-seq of total and AGO2-bound RNA samples; heatmap of miRNA expression changes found in DOX-treated versus vehicle-treated *TP53*^+/+^ HCT116 cells by small RNA-seq of total and AGO2-bound RNA samples; miRNA expression changes found in DOX-treated versus vehicle-treated *TP53*^−/−^ HCT116 cells by small RNA-seq of total and AGO2-bound RNA samples; histogram of changes in PAR-CLIP cluster abundance in DOX-treated versus vehicle-treated samples for HCT116 *TP53*^+/+^ cells. Data tracks in the figure render values of observed log-fold changes on a green (lower values) to red (higher values) scale. Ribbons in the circle *center* show miRNA-target interactions identified by analysis of chimera reads (i.e., ligation products, including part of the mature miRNA and part of its binding site sequence) from the PAR-CLIP experiments. (*C*) Result of motif analysis performed by using MEME (http://meme.nbcr.net/meme/) (Bailey et al. 2015) on the chimera target sites identified for let-7c. (*D*) Bar plots of the percentage of total chimera reads in DOX-treated samples compared to vehicle-treated samples.

We then extracted the miRNA–mRNA pairs specific for the activated (either more abundant or more associated with AGO2) and repressed (either less abundant or less associated with AGO2) miRNAs in response to DSBs to evaluate the miRNA–mRNA interaction subnetworks regulated by DSBs (Supplemental Fig. S10, bottom left and top right subnetworks). Since coordinated transcription and miRNA-mediated post-transcriptional repression enhances robustness of gene regulation in mammals (Tsang et al. 2007), we wanted to evaluate the global overall impact of miRNA regulation on gene expression in response to DSBs. To this end, we highlighted with green (down-regulation) or red (up-regulation) coloring the gene expression changes elucidated by RNA-seq for genes involved in the networks of miRNA–mRNA interactions derived from the PAR-CLIP data analysis (Supplemental Fig. S10). We used this approach to evaluate how these miRNAs coordinately affect genes that are up- or down-regulated following DSBs by other mechanisms, such as transcription. It appeared that miRNAs activated by DSBs targeted genes that were both up-regulated and down-regulated by the DNA damage itself (Supplemental Figs. S10, S12; Supplemental Table S5), indicating that after their activation, these miRNAs modulate the damage response through an intricate network of regulatory feedback and feedforward loops, supporting published models of miRNA regulation (O'Donnell et al. 2005; Tsang et al. 2007; Castellano et al. 2009). Interestingly, the subnetwork relative to targets of the let-7 family members (Supplemental Fig. S10, bottom right; Supplemental Table S5) was enriched for cell cycle and TP53-signaling pathways (Fig. 5A). This indicates that the TP53-dependent increase in binding of let-7 family members with AGO2 actively participates in the DSB response.

### let-7-mRNA chimeric reads from PAR-CLIP-seq are significantly more abundant after DOX treatment in TP53 wild-type cells compared with untreated cells

We then implemented a computational pipeline to identify unequivocal interactions between miRNAs and their targets by analyzing the miRNA–mRNA fusion products (chimeras) originating from endogenous ligase activity during the PAR-CLIP procedure (Fig. 5B; Supplemental Fig. S13; Supplemental Table S6; Grosswendt et al. 2014). Analysis of the distribution of the last nucleotide in both the miRNA and the target region of the selected chimera candidates confirmed a high prevalence of G (Supplemental Fig. S14A), as previously shown (Grosswendt et al. 2014). In addition, the target region of the identified chimeras substantially map to 3′ UTR regions, in contrast to the PAR-CLIP data that showed a similar frequency of miRNA interactions in 3′ UTRs and CDSs (Supplemental Figs. S8C, S14B). This indicates that miRNA–mRNA chimeras could be preferentially formed from 3′ UTR regions. We were unable to identify interactions for many abundant miRNAs, suggesting that the ligation between miRNAs and targets occurs more frequently for some miRNAs than others. We also observed that the sequences corresponding to the target regions obtained from the chimeras harbor a seed match for the cognate miRNA far more frequently than found in a set of random sequences (Supplemental Fig. S14C).

As expected, when we extended the sequences of the target part of the chimeras (to look for seed matches, as RNase digestion often shortens their length), we found that 5-mer matches complementary to the miRNA seed region occurred much more frequently than when adding random nucleotides (Supplemental Fig. S14C). Notably, 5-mer matches were more frequent than both 6- and 7-mers combined (Supplemental Fig. S14C), suggesting that 5-mers alone in the seed may be of great relevance for miRNA-target interaction.

Separately, and also as expected, 4-mer windows of complementarity to the target were most often enriched from the second position of 5′ end of the miRNA (Supplemental Fig. S15) again supporting the chimeras as bona fide target-miRNA hybrids.

Motif enrichment analysis of the chimera target sites indicated that those recognized by let-7 members tend to have more consistent perfect base-pairing to positions 3, 4, and 7 of the miRNA-seed region compared to other nucleotides in the seed, which show higher mismatch frequency (Fig. 5C; Supplemental Fig S14A,B), although different members of the let-7 family appear to have a slightly different recognition modes (Fig. 5D; Supplemental Fig S16A,B). Interestingly, by plotting the positional distribution of the best match for each motif identified as enriched in let-7c and let-7b target sequences, we found that the motif corresponding to the seed region was centered, with a peak between the center (0) and the −10 position of the analyzed data set of target sequences (Supplemental Fig. S16C,D). Notably, only let-7c targets were associated with U-rich motifs peaking next to the miRNA–mRNA “seed” interaction sites (Supplemental Fig. S13C, position between −30 and −20 of the graph), indicating that an additional RNA binding protein(s) might contribute to let-7c-mRNA target regulation. Importantly, the percentage of formed let-7c-5p-target chimeric reads significantly increased with DOX treatment (Fig. 5D), once again confirming that the TP53-dependent increase in interaction between let-7 family members and AGO2 functionally influences the DNA damage regulated miRNA-targetome.

## Discussion

In this study, we identified an unprecedented role for TP53 in interacting with AGO2 and modulating the association between a subset of miRNAs and AGO2 during the DNA damage response. Although a recent study demonstrated that hypoxia suppressed AGO2-mediated maturation of a group of miRNAs (Shen et al. 2013), to the best of our knowledge, our data represents the first evidence that an external stimulus could impact the association between specific miRNAs and AGO2, and this mechanism is unrelated to variations in their abundance. This indicates that an AGO2–miRNA interaction but not regulation of miRNA biogenesis is responsible. In particular, we found that the expression levels of specific let-7 family members, which usually act as tumor suppressor miRNAs negatively regulating cell cycle and differentiation processes (Boyerinas et al. 2010), are not differentially expressed following DSBs caused by DOX treatment. Rather, we observed modulation of the association of these and other miRNAs with the RISC complex. Moreover, we used state-of-the-art whole-genome techniques (including RIP-seq and PAR-CLIP-seq) and computational methods (such as the determination of miRNA–mRNA chimeras formed by endogenous ligase activity during the PAR-CLIP procedure) (Grosswendt et al. 2014) to evaluate the targeting networks of the DOX-regulated miRNAs. Using these techniques, we found that mRNAs that were more bound onto AGO2 following DOX treatment (indicative of miRNA post-transcriptional regulation mediated by the DNA damage response) were enriched for let-7 seed matches. We should mention that due to the selection of T-to-C conversions during PAR-CLIP analysis, sites lacking Us in the clusters of reads could have been missed.

Importantly, the let-7-mRNA chimeras that formed upon DOX treatment were significantly more abundant in DOX-treated samples in *TP53*^+/+^ but not *TP53*^−/−^ cells. These results indicate that a TP53-mediated increase of let-7 miRNAs' association with AGO2 following DOX treatment functionally influences the repression of their targets. Consistent with this finding, the let-7-regulated gene signature that we discovered showed functional enrichment in cell cycle and TP53-signaling pathways. Notably, we found that mismatches in the seed of let-7 members did not occur randomly, but were seen with much greater frequency at specific positions. Our finding that 5-mer seed matches represent a higher proportion of the miRNA-targetome than 6-mer and 7-mer matches combined indicates that this mode of miRNA binding could be sufficient for miRNA regulation more often than is currently appreciated.

Furthermore, we showed that various oncogenic mutant forms of TP53 decreased miRNA binding onto AGO2 rather than increasing it, as observed for wild-type TP53. This suggests an additional oncogenic mechanism through which these mutant TP53 variants function that implies loss of their capability to mediate let-7 association with AGO2 following DNA damage. Although the mechanism by which TP53 might regulate the association of certain miRNAs with AGO2 remains unknown, it is possible to speculate that TP53 interacting with AGO2 may modify the conformation of AGO2 or other RISC components and consequently change their affinity for a subset of miRNAs. Furthermore, the variable effect on miRNA–AGO2 associations that we observed using *TP53* mutants suggests that TP53's regulation of AGO2 binding may be dependent of its conformation, since these missense mutants can have local (R248W and R273H) or more comprehensive (R175H) conformational changes (Sigal and Rotter 2000). In conclusion, this report reveals a novel role for TP53 in regulating the DNA damage response, demonstrating an additional mechanism of miRNA regulation with relevance to cancer progression.

## Methods

Plasmid transfection, RT-qPCR, 3′ UTR luciferase reporter assay, bioinformatic processing of the RNA-seq data, and the statistical approaches as well as additional details are provided in the Supplemental Methods.

### Cell culture and DOX treatment

HCT116 *TP53*^+/+^ and *TP35*^−/−^, DLD1, and RKO cell lines were maintained in Dulbecco's modifed Eagle's medium or McCoy's medium supplemented with 10% FCS, 1% penicillin/streptomycin, and 2% glutamine. Cells were plated in 150-mm dishes at 50% confluence and incubated at 37**°**C in a humidified 5% CO_2_ incubator. They were then treated with DOX at a concentration of 0.2 µg/mL or equivalent volume of vehicle (ddH_2_O). After each treatment time point, dishes were placed on ice and medium was aspirated. Cells were washed twice with cold PBS, scraped, and centrifuged for 5 min at 1300 rpm. The supernatant was removed, and the cell pellet was processed for RNA using TRIzol reagent (Invitrogen) and/or protein extraction.

### Preparation of the RNA and small RNA libraries

RNA and small RNA libraries were produced using the Illumina TruSeq RNA Sample Prep Kit and Small RNA Sample Prep Kit, respectively (Illumina), according to the manufacturer's protocols. For both procedures, 200 ng RNA was used from the immunoprecipitation samples and 4 µg from the input samples. For the RNA sequencing (RNA-seq), paired-end sequences (reads) of 100 nt in length were then generated using a HiSeq 2000 instrument (Illumina). For the small RNA-seq, single-end reads of 50 nt in length were generated using a HiSeq 2000 instrument (Illumina).

### AGO2 RNA immunoprecipitation (RIP)

Cells were plated and treated with DOX at a concentration of 0.2 µg/mL or equivalent volume of vehicle for 24 h. Cells were washed in cold PBS, scraped, and then lysed with a buffer containing 0.5% Nonidet, 0.5 mM DTT, 20 mM Tris-HCL pH7.5, 150 mM KCL, 2 mM EDTA, 1 mM NaF, and inhibitors of RNases, proteases, and phosphatases. Ten percent of total lysate was removed and kept as the input samples and the remainder used for immunoprecipitation. Ten micrograms of anti-AGO2 (11A9, SAB4200085, Sigma-Aldrich) or anti-IgG (Sigma-Aldrich) antibodies were bound to sepharose beads in the presence of heparin. Precleared lysates were then incubated with the appropriate antibody-bound beads, and the immunoprecipitated proteins were then washed and incubated with DNase I in the presence of DNase buffer (Promega) followed by protease K (New England Biolabs) in the presence of 2× protease buffer (New England Biolabs). RNA extraction was then performed using phenol chloroform separation and ethanol/sodium acetate precipitation. RNA pellets were washed in ethanol and quantified using a Nanodrop.

### AGO2 PAR-CLIP

PAR-CLIP was performed as previously described (Hafner et al. 2010) with minor modifications. Briefly, a total of 400 million cells were used for each experiment. Each plate was treated with 4-Thiouridine (Sigma-Aldrich) at a final concentration of 100 µM 14 h prior to UV-crosslinking with 0.15 J/cm^2^ of 365-nm UV light with a Stratalinker UV Crosslinker (Stratagene). Cells were then scraped, lysed, and digested with RNase T1 (Fermentas) to a final concentration of 1 unit/µL. AGO immunoprecipitation of the lysate was then performed using Dynabeads Protein G magnetic particles (Invitrogen) and AGO antibody (Sigma) at a final concentration of 0.05 µg/µL. A second RNase T1 treatment was then performed using a final concentration of 10 units/µL. The RNA segments were then radiolabeled using 32P-γATP to a final concentration of 0.5 µCi/µL and T4 PNK to a final concentration of 1 unit/µL, and samples were then resuspended in 70 µL SDS-PAGE loading buffer, and SDS-PAGE was performed. The gel bands corresponding to AGO were cut for each sample and electroelution of the crosslinked RNA–protein complexes was then performed. Recovery of crosslinked target RNA fragments was then performed using phenol chloroform and ethanol extraction.

### Co-IP and RNase treatment

Cells were plated and treated with DOX at a concentration of 0.2 µg/mL or equivalent volume of vehicle for 24 h unless stated. After washing with cold PBS, cells were scraped following lysing with RIPA buffer. Ten percent total lysate was removed and kept as the input samples, and the remainder was used for immunoprecipitation following ImmunoCruz Optima Immunoprecipitation protocol, provided with Optima B and C (Santa Cruz Biotechnology). Preclearing Matrix B and C were used for optimal preclearing of the lysate (Santa Cruz Biotechnology). The following antibodies were used during the immunoprecipitation and immunoblotting: anti-TP53 (DO-1, sc-126; Santa Cruz); anti-AGO2 (ab32381, Abcam); anti-DDX5 (ab21696, Abcam); IgG (Santa Cruz).

To check for RNA dependence in TP53–AGO2 interaction, matrix-bound complexes were incubated with RNase A (Sigma) for 30 min at 37°C. Following incubation, samples were spun down and separated into supernatant- and bead-containing fractions and subjected to immunoblot analysis.

### Immunofluorescence

Following treatment with DOX, cells were fixed with 4% paraformaldehyde in PBS for 10 min, the excess of which was subsequently quenched with 0.5 M glycine in PBS for 20 min at room temperature (RT). For cell permeabilization, 0.3% Triton X-100 was added for 10 min. Next, cells were incubated with blocking buffer (5% goat serum, 1% BSA, 2% FCS in PBS) for 30 min followed by incubation with mouse monoclonal anti-TP53 antibody (1:100; sc-126, Santa Cruz) and rabbit polyclonal anti-AGO2 (1:250; ab32381, Abcam) for 1 h at RT. Cells were washed three times with 1% BSA and 2% FCS in PBS and incubated with Alexa 488 (anti-rabbit; Invitrogen) and 568 (anti-mouse; Invitrogen) for 2 h at RT. Next, cells were washed twice with 1% BSA and 2% FCS in PBS and incubated with 4% paraformaldehyde for 10 min at RT. Samples were subsequently washed twice with PBS and mounted using Mowiol 4-88 (Calbiochem) with DAPI. Slides were analyzed using a Leica TCS SP5 confocal laser-scanning microscope and analyzed using ImageJ software.

### Duolink proximity ligation assay (PLA)

After treatment with DOX, fixation, permeabilization, blocking, and primary antibody incubation steps were performed as described in the immunofluorescence section. Once cells were washed three times with 1% BSA and 2% FCS in PBS, incubation with PLA probes (i.e., anti-rabbit PLUS, anti-mouse MINUS PLA probes), ligation, amplification, and mounting of the coverslips were all performed according to the manufacturer's instruction (Sigma-Aldrich). Slides were imaged using a Leica TCS SP5 confocal laser-scanning microscope and analyzed using ImageJ software. The number of PLA foci was manually counted in a blind fashion (100 cells per each condition in the three independent experiments). Cyto-3 plasmic foci were defined as fluorescent spots that did not colocalize with the nuclei stained with DAPI. Statistical significance in the number of PLA foci was tested by using a two-tailed *t*-test.

## Data access

Sequencing data from this study have been submitted to the European Nucleotide Archive (ENA; http://www.ebi.ac.uk/ena), under accession numbers PRJEB3157 and PRJEB3233 for small and long RNAs sequencing data, and PRJEB3396, for PAR-CLIP data.
